# Catalytic Decomposition of H_2_O_2_ in the Aqueous Dispersions of the Potassium Polytitanates Produced in Different Conditions of Molten Salt Synthesis

**DOI:** 10.3390/molecules28134945

**Published:** 2023-06-23

**Authors:** Alexander Gorokhovsky, Natalia Morozova, Gleb Yurkov, Olga Grapenko, Alexander Kozinkin, Alexei Kozakov, Anatoliy Nikolskiy, Elena Tretyachenko, Andrey Semenov, Vitaliy Solodilov

**Affiliations:** 1 Department of Materials Chemistry and Technology, Yuri Gagarin State Technical University of Saratov, Polytekhnicheskaya 77, Saratov 410054, Russia; dlg2@yandex.ru (N.M.); trev07@rambler.ru (E.T.); 2 N.N. Semenov Federal Research Center of Chemical Physics, Russian Academy of Sciences, Kosygina 4, Moscow 119991, Russia; cemen9856@gmail.com (A.S.); vital-yo@yandex.ru (V.S.); 3 Research Institute of Physics, Southern Federal University, pr. Stachki 194, Rostov-on-Don 344090, Russia; grapenko@sfedu.ru (O.G.); kozinkin@sfedu.ru (A.K.); atkozakov@sfedu.ru (A.K.); anikolskiy@sfedu.ru (A.N.)

**Keywords:** layered titanates, structural features, H_2_O_2_ decomposition, catalysis, kinetics and mechanism

## Abstract

It is shown that the potassium polytitanate powder (PPT) synthesized at 500 °C via the treatment of powdered TiO_2_ (rutile) in molten mixtures of KOH and KNO_3_ is a cheap and effective catalyst of H_2_O_2_ chemical decomposition in aqueous solutions. At the same time, the PPT catalytic activity strongly depends on the [TiO_2_]:[KOH]:[KNO_3_] weight ratio in the mixture used for the synthesis, increasing with [KNO_3_] in the order of PPT (30:30:40) < PPT (30:50:20) < PPT (30:70:0). The obtained results are explained by increased [Ti^3+^] in the PPT structure (XPS data), which is grown in this order from 0 to 4.0 and 21.9 at.%, respectively, due to the reduced oxidation activity of the melt used for PPT synthesis. The mechanism of the autocatalytic process taking place in the PPT-H_2_O_2_-H_2_O system is analyzed. Taking into account the data of FT-IR spectroscopy, it is assumed that the increased catalytic activity of the investigated materials is related to the increased surface concentration of the Ti^4+^-O(H)-Ti^4+^ groups, formed from the Ti^3+^-O(H_3_O^+^)-Ti^4+^ clusters and further transformed into Ti-O-O-H catalytic centers. Some possible applications of the PPT-H_2_O_2_-H_2_O catalytic system, including the oxidation processes of green chemistry and photo-catalysis, are discussed.

## 1. Introduction

Organic synthesis and water pollutant decomposition using H_2_O_2_ as an oxidant are excellent green oxidation processes due to their high efficiency and the lack of dangerous by-products [1,2]. However, H_2_O_2_ can be an ideal, waste-avoiding oxidant only when it is used in a controlled manner without organic solvents [3]. At the same time, H_2_O_2_ can undergo radical decomposition into H_2_O and ½ O_2_ in a violent exothermic reaction, which can lead to an explosion. To reduce the decomposition temperature of H_2_O_2_, some transition and rare-earth metals, as well as their oxides, can be used as catalysts. Oxidation reactions take place on their surface with the participation of molecular oxygen and peroxides and contribute to the oxidation of the adsorbed organic compounds, and the reduced form of the catalyst is subsequently re-oxidized by O_2_ or H_2_O_2_ [1,2].

Nowadays, a number of heterogeneous catalysts used for oxidation processes in the presence of H_2_O_2_ have been developed [4,5]. Many catalytic systems based on noble metals, as well as tungsten, manganese, rhenium, and titanium compounds, have been reported for their use the oxidation of different organic compounds using hydrogen peroxide [1,2,4]. However, most of them are unstable and have limited recyclability. Furthermore, their use is accompanied by a very intensive release of molecular oxygen and the violent (up to 100 °C) heating of the system. That is why the search for new efficient and cheap catalysts, which could support H_2_O_2_ decomposition under the reaction conditions at temperatures of less than 40–45 °C (beginning of the H_2_O_2_ pyrolysis), is a very urgent problem.

Whereas TiO_2_ is one of the most promising, relatively cheap, and stable catalysts and photocatalysts for oxidation processes [5,6,7,8], there is a great interest in the use of TiO_2_-based compounds and different titanates for these purposes.

It is known that photochemical oxidation using a catalytically active complex, H_2_O_2_-TiO_2_, provides the oxidation of organic substances when irradiated with solar light [9,10,11,12,13]. The product of their interaction is sensitive to visible light due to the formation of the peroxo-complex (Ti^4+^OOH) on the TiO_2_ surface [10]. However, the mechanism of this process is very poorly studied, although it is known that the oxidative activity of titanium peroxide depends on the crystalline structure of the parent TiO_2_ and the pH of the aqueous solutions used for the synthesis [9,11,12]. An increase in the pH value, associated with increased hydroxylation of the TiO_2_ surface, favors catalytic activity. However, the reaction of molecular oxygen formation onto the surface of powdered TiO_2_ upon their interaction with H_2_O_2_, leading to the formation of TiOOH surface groups (active catalytic centers), has a very low rate due to the relatively low content of the surface TiOH groups [13].

In this regard, oxidation processes involving nanoscale TiO_2_ powders, characterized by relatively large specific surface area, have been intensively studied over the past decade in relation to the production of water-soluble forms of titanium oxide peroxide via the sol–gel technique using peroxotitanic acid as an intermediate [14]. The addition of alkalis can induce increased activity in the obtained product. However, very intensive heating of the sol prevents the polymerization of the synthesized peroxotitanic acid and promotes obtaining this catalyst in a water-soluble form. Such water-soluble peroxo-titanium complexes have been used as homogeneous catalysts for oxidation [15,16]. However, the use of homogeneous oxidation catalysts meets the problem of the subsequent separation of these catalysts from the obtained water-soluble products.

As a result, producing highly efficient mesoporous titanates based on the peroxide form of TiO_2_ attracts considerable attention [17,18,19]. However, the sol–gel synthesis of such heterogeneous catalysts is complicated and expensive, which is why several ceramic micro- and mesoporous heterostructured materials based on the TiO_2_-SiO_2_ system have also been developed as an alternative to porous catalysts based on titanium oxide peroxide [20]. In the catalysts of this group, titanium species are incorporated into the mesoporous silicate structure. Nevertheless, the low content of TiO_2_ in such catalysts (1.5–4.5 wt.% [18]) and the poor accessibility of the peroxide sites for organic molecules and H_2_O_2_ significantly limit their efficiency [21].

Taking into account the above mentioned reasons, it was proposed that the potassium polytitanates (PPTs) produced via hydrothermal [22,23] or molten salt [24] synthesis and characterized with high hydroxylation of the surface, large interlayer distance, the tendency to form large agglomerates, and the basic character of their aqueous dispersions, could be considered to be a new type of heterogeneous catalyst destined for oxidation processes in aqueous media.

The purpose of this work was to investigate the catalytic activity of the potassium polytitanate powders, which were produced in the molten KOH-KNO_3_ mixtures characterized by various oxidation activities and estimate the influence of this factor on the kinetics of H_2_O_2_ decomposition in the presence of these kinds of PPTs. On the other hand, the aim of this study was to search for experimental conditions that ensure the occurrence of redox catalytic reactions in the PPT-H_2_O_2_-H_2_O system, considered as the oxidizing medium that provides a constant rate of H_2_O_2_ decomposition without the occurrence of sharp temperature jumps.

## 2. Results and Discussion

### 2.1. PPTs Characterization

**Figure 1:**
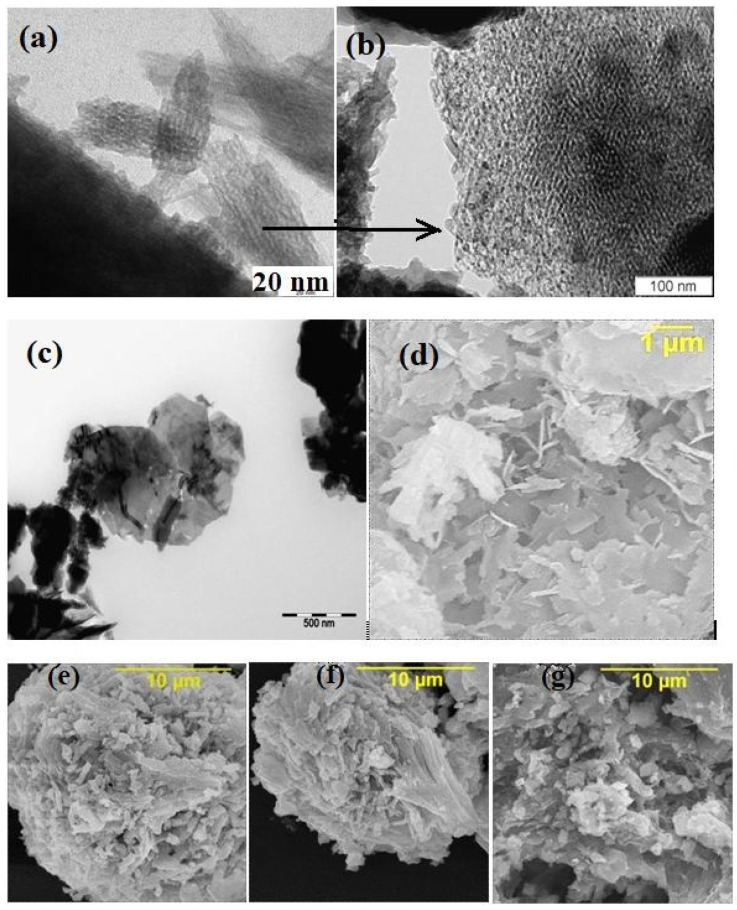
Typical electron images of the potassium polytitanate powders (individual (**a**), agglomerated (**b**,**c**) and aggregated (**d**–**g**) particles); PEM (**a**–**c**), PEM of high resolution (**b**) and SEM (**d**–**g**) PPT(30-30-40) (**a**–**c**,**g**), PPT(30-70-0) (**e**), PPT(30-50-20) (**f**), and PPT(10-3-87) (**d**).

**Figure 2:**
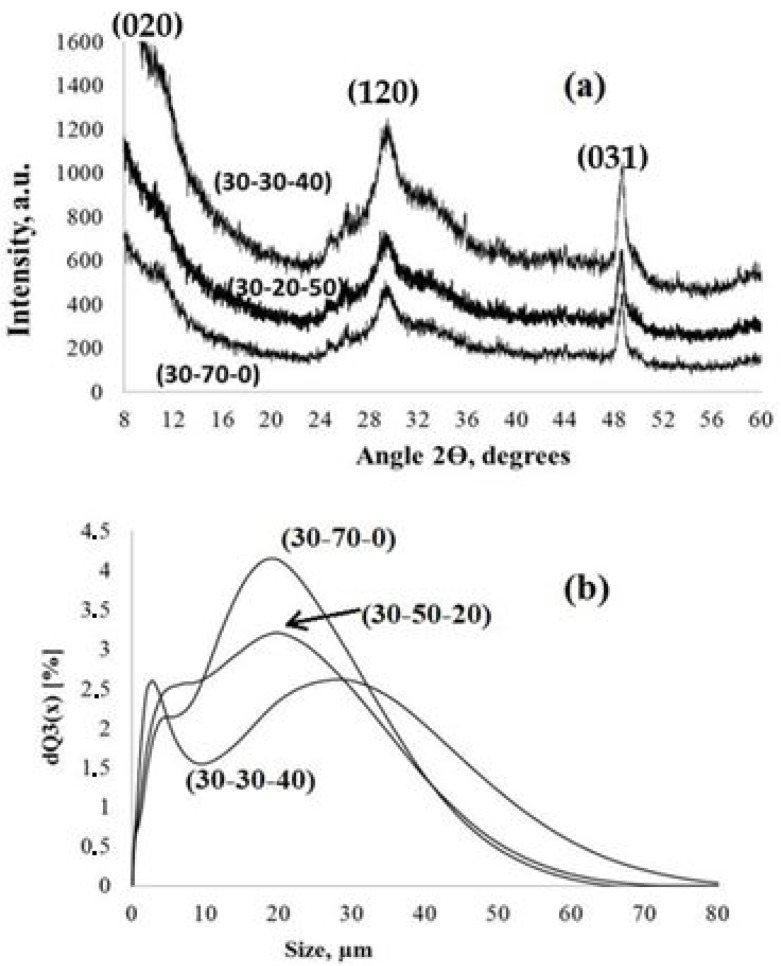
XRD patterns (**a**) and particle size distribution (**b**) of the PPT powders synthesized under different conditions (noted in Table 2).

All of the parent PPT powders, independent of the conditions used for synthesis, had similar TiO_2_/K_2_O molar ratio equal to 4.0 ± 0.1 (EDS data) and consisted of nanoscale flakes (Figure 1a), forming quasi-amorphous platy particles (Figure 1b,c) 100–800 nm in diameter and thicknesses of 20–30 nm, aggregated in porous agglomerates of 2–4 µm in diameter (Figure 1d–g) and large-scale aggregates with an average size of 20–30 µm (Figure 2). All of the aggregates have a porous structure, supporting the availability of the PPT particles’ surface-active centers for the reagents coming from the solution in the aqueous dispersions. All of the PPT powders have significantly large specific surface areas (71.3–79.6 m^2^/g, Table 1), which slightly decrease with increased [KNO_3_]/[KOH] ratio in the melts used to synthesize these products (Table 1).

The XRD data also confirm the quasi-amorphous structure of the obtained PPT powders. The XRD patterns only have weak and wide reflections, typical for lepidocrocite-like crystalline structures [25].

The size of the PPT particles was estimated using the XRD patterns in accordance with the Selyakov–Scherrer equation:

*d*_*XRD*_ = (*K* × *λ*)/(*B* × *Cosθ*)

*(d_XRD_* is an average crystallite size; *λ* is the wavelength of the copper Kα line, 0.15406 nm; *B* is a half-width of the (*hkl*) reflection; *θ* is the diffraction angle; and *K* is a constant), differs for the reflections recognized at angles of 2θ of 29.6° and 48.8°. For the reflection at 48.8° (*031*), the calculated size of the crystallites is 22.8 ± 0.2 nm for all of the PPT powders and corresponds to the TEM data on the diameter of PPT nano-flakes (Figure 1a,b). The size of the crystallites calculated using the Selyakov–Scherrer equation for the refection at 29.6° (*120*) is 9 ± 1 nm and could be related to the thickness of these particles (Figure 1d). It is also important that the low angle 2θ reflection (020), which is typical for lepidocrocite-like structures, is very wide and shifted to a range of less than 8° (measurement limit for the used equipment), indicating large (up to 1 nm) interlayer distance [22].

In any case, it is known that the result of particle size estimation derived from the Selyakov–Scherrer formula is approximate due to the fact that “crystallite size” is not synonymous with “particle size”, while X-ray diffraction is sensitive to the crystallite size inside the particles [26]. In addition, it is known that K ≈ 0.9 is only for the spherical crystals with cubic unit cells and can acquire other values for one-dimensional and two-dimensional crystals [27]. The last is important considering the platy shape of the PPT nanoparticles (Figure 1a). In the structural aspect, potassium polytitanate is a disordered network of quasi-two-dimensional nanosized clusters (domains), which can have a regular structure in some parts of the particles. That is why the obtained data can only be used as semi-quantitative.

Thus, to specify the differences that take place among the PPT powders obtained in various experimental conditions, it is necessary to compare the other structural characteristics of these substances.

**Figure 3:**
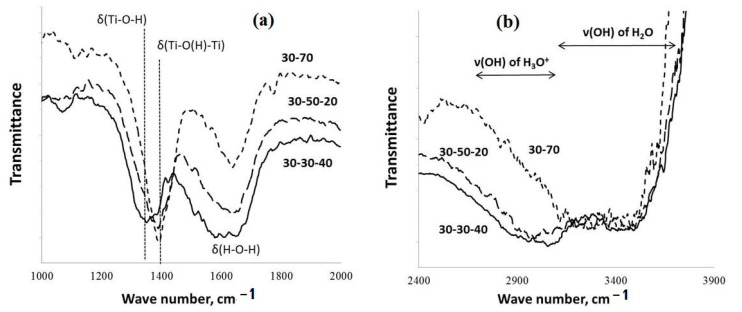
FT-IR spectra of the PPT powders obtained under different experimental conditions: (**a**) wave range from 1000 to 2000 ${cm}^{-1}$; (**b**) wave range from 2400 to 3900 ${cm}^{-1}$.

**Figure 4:**
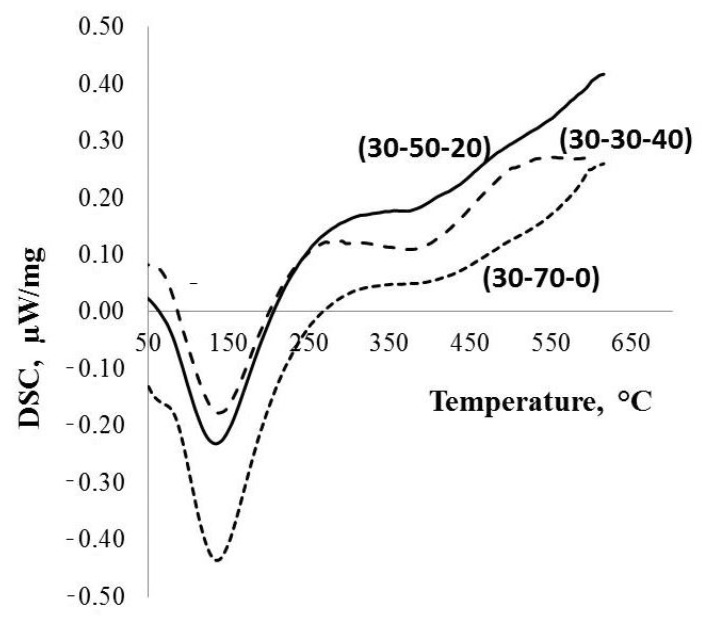
DSC and TGA data for the parent PPT powders produced under different conditions.

The FT-IR spectra and DSC data corresponding to various investigated kinds of potassium polytitanate produced via molten salt synthesis are reported in Figure 3 and Figure 4.

The data of FT-IR spectroscopy indicate (Figure 3) that the increased contents of KOH in the molten KOH-KNO_3_ mixtures used for the PPT synthesis support increased [(Ti-O(H)-Ti)] in comparison with [Ti-O-H]; the maximum of the absorption band at 1350 cm^−1^ (attributed to δ(H-O-H)) shifts to 1400 cm^−1^ (attributed to δ(Ti-OH) and δ(Ti-O(H)-Ti)) [28,29,30].

Furthermore, such synthesis conditions promote the reduced intensity of the absorption band related to δ(H-O-H) (1640 cm^−1^). At the same time, a decrease in the [KNO_3_]/[KOH] ratio provides reduced absorption in the range of frequencies corresponding to the symmetric and asymmetric stretching vibration of ν(O-H) in the hydronium ions (H_3_O^+^) (2600–3200 cm^−1^), whereas, the intensity of the absorption band at 3300–3700 cm^−1^ (the stretching vibrations of ν(O-H) in the H_2_O molecules, in accordance with [28,29,30]) almost does not change.

**Table 1:** Structural characteristics of the PPT powders synthesized under different experimental conditions. S: specific surface area. [H_2_O]_PA_ and [H_2_O]_CA_: contents of physically and chemically adsorbed water (TGA).

No	Reference	$\frac{\left[KNO_{3}\right]}{\left[KOH\right]}$	$\frac{\left[KOH\right]}{\left[TiO_{2}\right]}$	S, m^2^/g	Contents * of Ti^n+^, at.%			[H_2_O]_PA_(25–390 °C), wt.%
					Ti^4+^	Ti^3+^	Ti^2+^	
1	(30-70-0)	0	2.33	79.6	74.8	21.9	3,3	8.5
2	(30-50-20)	0.4	1.67	76.3	83.9	16.1	-	7.9
3	(30-30-40)	1.33	1	72.9	96.0	4.0	-	6.0
4	(10-3-87)	29.0	0.3	71.3	100	-	-	2.5

**Figure 5:**
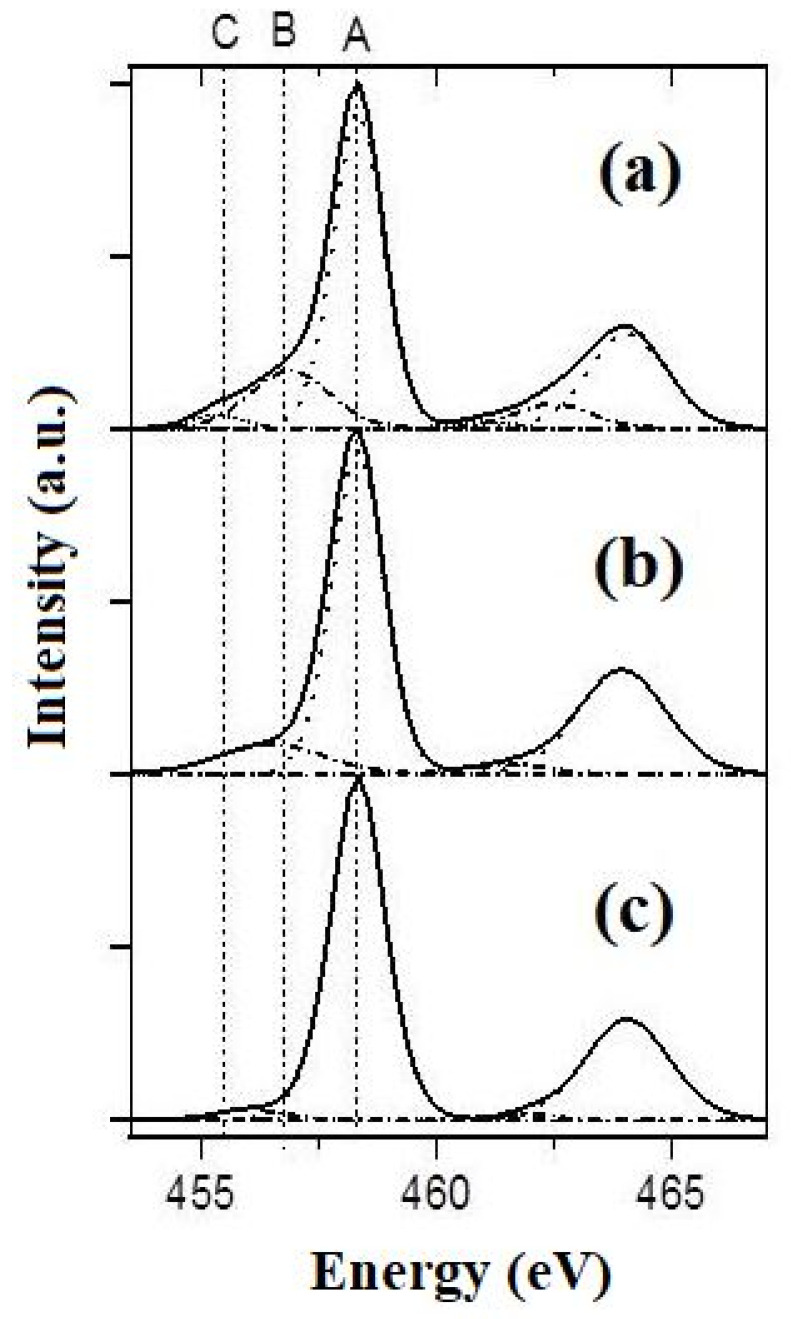
XPS spectra in the energy range of Ti2p for the specimens of PPT (30-70-0) (**a**), PPT(30-50-20) (**b**), and PPT (30-30-40) (**c**). A: 2p(3/2) Ti4+; B: 2p(3/2) Ti3+; C: 2p(3/2) Ti2+.

* XPS data in accordance with the intensities of A, B and C bands (Figure 5).

**Table 2:** The chemical compositions of raw material mixtures used to produce different kinds of potassium polytitanate.

N No	Reference of the Obtained PPT Product	Synthesis Conditions				
		Content of the Component, wt.%			Weight Ratio of the Components	
		TiO_2_	KOH	KNO_3_	[KNO_3_]/[KOH]	[KOH]/[TiO_2_]
1	(30-70-0)	30	70	0	0	2.33
2	(30-50-20)	30	50	20	0.4	1.67
3	(30-30-40)	30	30	40	1.33	1
4	(10-3-87)	10	3	87	29	0.3

The DTA data (Figure 4) indicate that all of the investigated types of the PPT are characterized by the presence of different forms of water: (1) physically adsorbed, corresponding to the endothermic peak at ~150 °C, and (2) chemically adsorbed, related to a wide endothermic peak with a maximum at about of 390 °C. In addition, the quantity of physically adsorbed water decreases in the order of PPT(30-70-0), PPT (30-50-20), and PPT (30-30-40) (Table 1).

The XPS spectra (Figure 5) indicate the presence of various forms of titanium (Ti^4+^, Ti^3+^, and Ti^2+^) in the structure of the powders studied.

The broad Ti2p3/2 peak was decomposed into three components of the Gaussian form. The ones at 455.3 and 457.2 eV are attributed to Ti(II) and Ti(III), respectively, while that at 458.6 eV is indexed to Ti(IV) in accordance with the earlier published results and the calibration XPS spectra of PbTiO_3_ [31]. The contents of these structural species were estimated using the integral intensities of the corresponding peaks (Table 1). These data indicate that the increased oxidative activity of the molten KOH-KNO_3_ mixtures, which are used to produce PPTs, leads to decreased concentrations of Ti^3+^ and Ti^2+^ species in the synthesized PPT powders.

The decomposition of the O1s X-ray photoelectron spectra into the components shows at least three oxygen states in PPT powders. The ground state (83%), with energy E = 530.0 eV, corresponds to oxygen in TiO_6/2_ octahedra, similar to those previously reported for TiO_2_ (E = 530.3–530.6) [32]. The remaining states with energies of 531.7 and 533.2 eV can correspond to oxygen in the structure of the surface Ti-O-H groups and adsorbed water, respectively.

### 2.2. Kinetic Experiments

**Figure 6:**
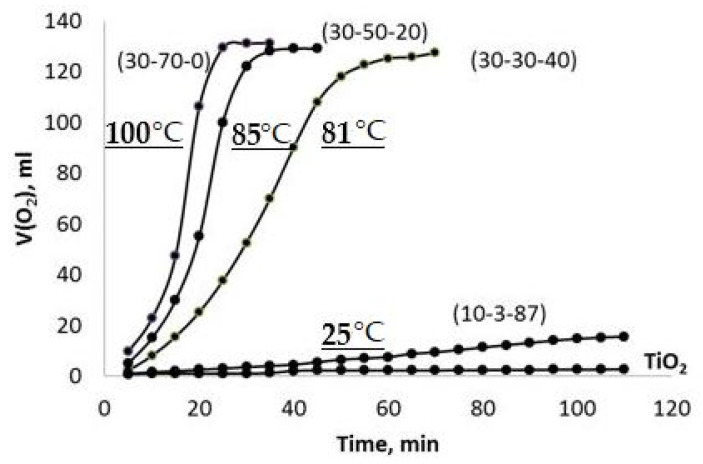
Kinetics of the chemical interaction of different types of PPT with 35% H_2_O_2_ aqueous solution (V(O2)-t plots, Series 1). The maximum values of the temperature, achieved in the systems during the reaction, are marked as curves.

A representative set of the observed V(O_2_)-t plots obtained using the PPT powders synthesized under different conditions (Series 1) are given in Figure 6. These plots represent sigmoid curves, which are typical for autocatalytic reactions [33]. The chemical processes taking place in the system of PPT-H_2_O_2_-H_2_O at the start proceed slowly (the induction period) because of the relatively low content of catalytic centers; however, the rate of reaction increases progressively as the reaction proceeds as the number of catalytic centers increases and slows down as the H_2_O_2_ concentration decreases.

It is possible to note that all the investigated potassium polytitanates are much more active in the chemical interaction with H_2_O_2_ in comparison with TiO_2_ nanopowder in spite of the agglomerated structure of the PPT particles.

Moreover, decreased [KNO_3_]/[KOH] and increased [KOH]/[TiO_2_] ratios in the raw materials mixtures, which are used to produce potassium polytitanate, promote more rapid chemical interactions in the obtained PPT powders with H_2_O_2_ aqueous solution, accompanied and supported by the increased heating of the dispersion. Furthermore, an increase in the [KNO_3_]/[KOH] ratio of up to 29, PPT(10-3-87), drastically reduces the rate of interaction (heating of the reaction mixture) and approximates the obtained effect almost to the case of TiO_2_ used as a catalyst.

Since the oxidation system of PPT-H_2_O_2_-H_2_O is of interest for use under conditions of stable and low temperatures, the second series of experiments was carried out to meet this requirement via the dilution of the parent 35% H_2_O_2_ aqueous solution with distilled water (Series 2).

**Figure 7:**
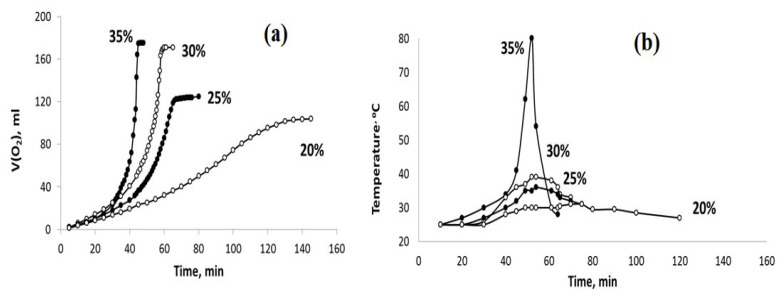
The sets of the observed V(O2)-t (**a**) and T–t (**b**) plots obtained using the PPT (30-70-0) catalyst and the H_2_O_2_ aqueous solutions of different concentrations (marked in %).

**Figure 8:**
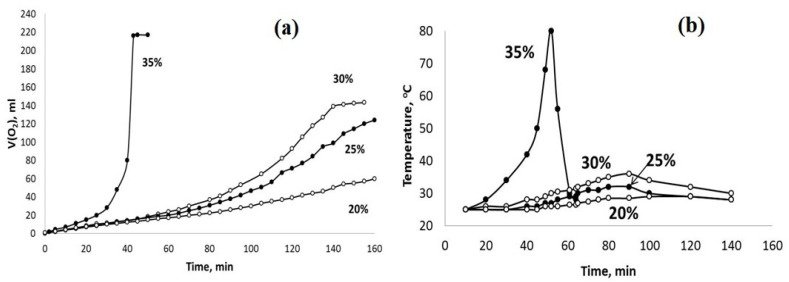
The sets of the observed V(O2)-t (**a**) and T–t (**b**) plots obtained using the PPT (30-30-40) catalyst and the H_2_O_2_ aqueous solutions of different concentrations (marked in %).

The influence of different admixtures of H_2_O in the aqueous dispersions of PPT (30-30-40) and PPT (30-70-0) on the kinetics of their interaction with H_2_O_2_ are reported in Figure 7 and Figure 8.

The obtained results indicate that the dilution of the hydrogen peroxide aqueous solution with water reduces the rate of H_2_O_2_ chemical decomposition and inhibits the heating of the dispersions obtained. However, these effects are more pronounced for the PPT (30-70-0) powder. On the other hand, it is important to note that the rate of oxygen generation can be approximately constant at a certain concentration (dilution) of H_2_O_2_ aqueous solutions.

### 2.3. Mechanism of the Catalytic Decomposition of H_2_O_2_

The obtained data indicate that the catalytic properties of PPT powders in the H_2_O_2_ decomposition process strongly depend on the experimental conditions used to produce these powders. All of the investigated types of PPTs are characterized by similar morphologies (agglomerated quasi-amorphous platy particles, Figure 1 and Figure 2), similar specific surface area (73–79 m^2^/g), and chemical composition ([TiO_2_]/[K_2_O] ≈ 4) (Table 1); however, their catalytic activity decreases in the number: PPT(30-70-0) > PPT (30-50-20) > PPT (30-30-40).

In accordance with [34,35], the peroxide groups appear on the surface of TiO_2_ under dark conditions as a result of Ti-O-H interaction with H_2_O_2_ molecules.

Ti-O-H (Ti-O(H)-Ti) + H_2_O_2_ → Ti-O-O-H (Ti-O(OH)-Ti) + H_2_O (1)

Ti-O-O-H (Ti-O(OH)-Ti) + H_2_O_2_ → Ti-O-H (Ti-O(H)-Ti) + O_2_↑ + H_2_O (2)

Thus, it can be argued that the increased oxidation activity of the KOH-KNO_3_ melts promotes the increased hydroxylation of the surface in the PPT powders produced. In any case, a washing of the parent PPT powders with water is accompanied by the ion-exchange process:

(K^+^)_S_ + (H_3_O^+^)_V_ = (H_3_O^+^)_S_ + (K^+^)_V_ (3)

The following behavior of the hydronium ions depends on the contents of Ti^3+^. In the PPTs with relatively low [Ti^3+^], such as PPT (30-30-40), the H_3_O^+^ ions stabilize in the interlayer space of PPT particles (Figure 4), whereas in the PPTs with higher concentrations of Ti^3+^, PPT (30-50-20) and PPT (30-70-0), it is possible to assume the following transformation:

Ti^3+^-O-Ti^4+^ + H_3_O^+^ = Ti^3+^-O(H_3_O^+^)-Ti^4+^ = Ti^4+-^O(H)-Ti^4+^ + H_2_O (4)

accompanied by the transfer of electrons from Ti^3+^ to H_3_O^+^ and an increased concentration of Ti-O(H)-Ti groups (Figure 3). The last provides increased catalytic activity in the order of PPT (30-30-40) < PPT (30-50-20) < PPT(30-70-0), corresponding to increased [Ti^3+^].

**Figure 9:**
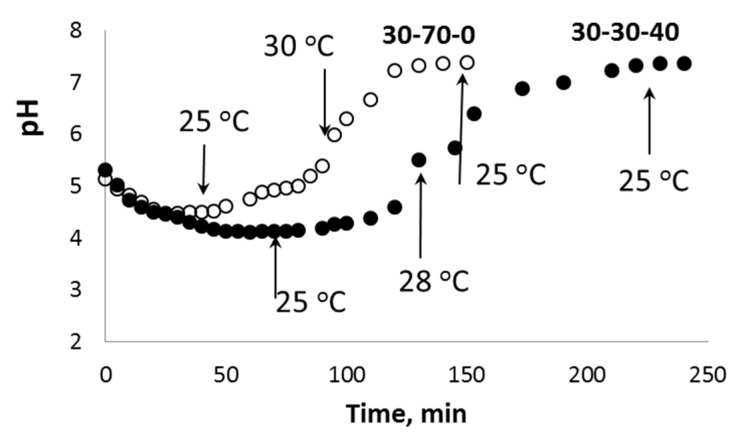
Change in the pH value during the interaction between aqueous dispersions of different PPT powders (pH0 = 10.7) and 20% H_2_O_2_ aqueous solution (pH0 = 3.8). The temperatures of the dispersion are marked with arrows.

The high acidity of H_2_O_2_ aqueous solutions provides additional K^+^ ↔ H_3_O^+^ ion exchange during the initial stage of the chemical processes in the PPT-H_2_O_2_-H_2_O system. Taking into account the porous structure of the aglomerated PPT powders, this process does not occur instantly and requires a certain induction period, which is clearly observed in the kinetic covers of oxygen generation (Figure 7 and Figure 8). During this initial stage, the pH value of the dispersion decreases from pH = 10.7 (PPT aqueous dispersion) to pH = 4–5 (PPT dispersion in the H_2_O_2_ aqueous solutions) (Figure 9). Increased contents of Ti-O(H)-Ti species promote the processes of (1) and (2). The Ti-O-OH (Ti-O(OH)-Ti) groups formed in process (1) are considered to be active catalytic sites, which, according to [36], lead to the yellow coloration of the catalyst. That is why the rate of transformation of white-colored potassium polytitanates into yellow-colored powders strongly depends on the presence of Ti^3+^ in their structure. Furthermore, the PPT-H_2_O-H_2_O_2_ system can be considered to be an autocatalytic one characterized by the formation and regeneration of catalytic centers (processes (1) and (2)) during the H_2_O_2_ decomposition.

Thus, the mechanism of H_2_O_2_ decomposition in the PPT-H_2_O_2_-H_2_O system is similar to the one previously considered for the MnO_2_-H_2_O_2_-H_2_O system [37,38] but also includes the generation processes of the additional catalytically active surface centers. The PPT catalysts characterized with higher contents of [Ti^3+^] have improved catalytic activity due to the increased surface concentration of Ti-O(H)-Ti groups. The proposed mechanism is similar to the conclusion reported in [39], where heterogeneous H_2_O_2_ catalytic decomposition was investigated on various metal foils.

Hydrogen peroxide decomposition has a strong exothermal effect. That is why an increase in the reaction rate (auto-acceleration) causes an increase in heat release, and the reaction develops according to the thermal explosion scheme (Figure 6, Figure 7 and Figure 8 concentrated H_2_O_2_ solutions). However, in accordance with a well-known work of N.N. Semenov [40], the thermal effect of the explosive reaction of the H_2_O_2_ catalytic decomposition can be balanced by the heat removal process related to the heating of the entire reaction system, including the reactor and H_2_O. In our case, this effect takes place due to the dilution of the H_2_O_2_ aqueous solution (Figure 7 and Figure 8).

In any case, the agglomerated potassium polytitanate powders, formed by particles characterized with relatively high contents of Ti^3+^, exhibit good catalytic properties in the decomposition of H_2_O_2_ aqueous solutions. The potassium polytitanates have a slightly lower catalytic activity in this process in comparison with Cu–Ce–O composite oxides, LaFe_x_Ni_1−x_O_3+δ_ and Au-modified carbon nanotubes [41,42,43], but the last is much more expensive. Some other relatively cheap catalysts, such as MnO_2_, and ZnO-doped cobaltic oxide [37,44], represent the powders with low specific surface area; manufacturing the honeycomb and mesoporous composite materials based thereon [20,38] solves this problem; however, it significantly increases due to the cost of such materials.

Thus, the PPT powders, produced via molten salt synthesis and characterized with high [Ti^3+^], can be considered new promising low-cost catalysts of H_2_O_2_ decomposition in aqueous solutions and may be recommended for use in the oxidation processes of green chemistry and in the systems generating O_2_ with a controlled rate, i.e., for medical purposes (oxy-therapy). The powdered products obtained as a result of the PPT chemical interaction with H_2_O_2_ aqueous solutions (peroxide form of potassium polytitanate) have a yellow color and could be potentially considered prospective photocatalysts that can be active in the visible range of solar radiation and could be destined for water purification or antioxidant applications, similar to [45].

## 3. Materials and Methods

### 3.1. PPT Synthesis

The potassium polytitanate powders (PPTs) were synthesized via the molten salt method, as earlier described in [24], using TiO_2_ (rutile), KOH, and KNO_3_ as raw materials. The batch (100 g of solids) was mixed with 60 mL of distilled water in the Al_2_O_3_ crucible; the obtained dispersion was agitated to dissolve KOH and KNO_3_ and further heated and thermally treated at 500 °C for 2 h in the electric furnace. The chemical composition of the batches is reported in Table 2.

These compositions were selected to form the raw material mixtures characterized with different contents of oxidizer using varied [KNO_3_]/[KOH] ratios at the same [TiO_2_] (No 1–3). It was proposed that the content of oxidizer could influence the structural features of the final PPT product. Batch No 4 was used as a reference one, which allowed obtaining the PPT product in the presence of a minimal content of KOH (maximal oxidation activity of the molten salt medium) [24,46]. In addition, the TiO_2_ nanopowders (Degussa P25, rutile, 20 nm) were used to compare the catalytic activity of the investigated materials.

The synthesized potassium polytitanates were carefully washed with distilled water three times to obtain dispersions characterized with pH = 10.7 ± 0.1, further filtrated (Whatman paper No. 40) and dried at 50 °C for 4 h in the oven.

### 3.2. Materials Characterization

The synthesized particles morphology was investigated via electron microscopy: TEM (Carl Zeiss Libra 120, WKα, 80 kV, Germany), high-resolution TEM (Jeol JEM-1011, 80 kV, Japan), and SEM (Jeol 5800LV, Japan and ASPEX Explorer, 20 kV, USA).

The chemical composition of the PPT specimens was characterized via wavelength dispersive X-ray fluorescence (BRA 135 F, Russia). The phase composition of the PPT powders was investigated using XRD analysis (ARL X’TRA diffractometer, CuK_α_, 40 kV, 100 mA, Switzerland).

X-ray photoelectron spectroscopy (XPS; VG Scientific ESCALAB 250; AlKa, 15 kV, 20 mA, UK) was used to estimate the contents of Ti in different valence states. All binding energy values (eV) were determined with respect to the C1s line (285.0 eV) originating from adventitious carbon, and the positions of the peaks were determined with an accuracy of ±0.2 eV. The resolution of the spectra was estimated to be 0.6 eV.

The particle size distribution in the powdered PPT specimens was studied using an Analysette 22 Microtec Plus laser analyzer, Germany. The measurements were carried out with a preliminary ultrasonic treatment of the powders in a liquid medium (distilled water) for 30 s at 10 W.

The specific surface area (m^2^/g) was determined via BET-analysis of N_2_ adsorption isotherms determined on the test specimens at liquid nitrogen temperature. Prior to exposure to the adsorptive molecules, the PPT specimens were outgassed at 100 °C and 10^−6^ Torr for 4 h.

The IR spectra of the PPTs were recorded with an FT-801 IR-Fourier spectrometer, Russia, using compressed tablets of the PPT-KBr mixtures at a weight ratio of 1:20.

The thermal analysis was performed by DSC (NETZSCH STA 449 F3, Germany) in an atmosphere of inert gas (Ar) at a scanning rate of 10 K/min.

### 3.3. Kinetic Measurements

To investigate the kinetics of the chemical interaction of the PPT powders with the hydrogen peroxide aqueous solutions, 0.05 g of the potassium polytitanate was mixed with 1.65 g of the 35% H_2_O_2_ aqueous solution (Carl Roth GmbH). The mixtures were prepared at room temperature (24 ± 1) °C, and all the experiments (Series 1) were carried out in the absence of light to prevent the photochemical degradation of H_2_O_2_ molecules.

Another series of experiments (Series 2) was prepared using the same quantity (weight) of the PPT powdered specimens (0.05 g) and aqueous solutions with the same (0.585 g) quantity of H_2_O_2_ but different concentrations obtained with distilled H_2_O admixtures (1.0; 1.5; 2.0; 2.5 g).

The reaction kinetics followed through the determination of the volume of oxygen released (V_t_) as a function of time using a homemade gasometer similar to that described earlier [47]. The temperature of the dispersion was controlled by thermocouple with a systematic error of ±0.5 °C.

## 4. Conclusions

1. The potassium polytitanate (PPT) produced via the treatment of TiO_2_ powder in the molten KOH-KNO_3_ mixtures is a new catalyst of the H_2_O_2_ decomposition in aqueous solutions.
2. The potassium polytitanates synthesized using various [KNO_3_]/[KOH] ratios, in spite of the same chemical composition and similar quasi-amorphous layered structure, are characterized by different catalytic activities.
3. The main cause of their different catalytic activity is related to various contents of TI^4+^ and Ti^3+^ in the PPT particles formed in the media characterized by different oxidizing activity.
4. An increase in the [KNO_3_]/[KOH] ratio in the molten mixture used for the treatment supports the higher oxidation activity of the melt and reduced [Ti^3+^] in the final product.
5. Increased contents of [Ti^3+^] promotes the transformation of the adsorbed hydronium ions in the additional surface Ti^4+^-OH (Ti^4+^-O(H)-Ti^4+^] groups, which interact with the adsorbed H_2_O_2_ molecules, forming Ti-O-O-H catalytic centers and increasing the rate of H_2_O_2_ decomposition.
6. The regulated dilution of PPT-H_2_O_2_-H_2_O dispersion by water allows obtaining the system characterized with the constant rate of the hydrogen hydroxide decomposition as well as high and stable oxidizing conditions.

## Data Availability

Not applicable.
